# Cleft palate repair and variations

**DOI:** 10.4103/0970-0358.57197

**Published:** 2009-10

**Authors:** Karoon Agrawal

**Affiliations:** Department of Plastic Surgery, Jawaharlal Institute of Postgraduate Medical Education and Research (JIPMER), Pondicherry- 605 006, India

**Keywords:** Cleft palate, Palate repair, Palatoplasty

## Abstract

Cleft palate affects almost every function of the face except vision. Today a child born with cleft palate with or without cleft lip should not be considered as unfortunate, because surgical repair of cleft palate has reached a highly satisfactory level. However for an average cleft surgeon palatoplasty remains an enigma. The surgery differs from centre to centre and surgeon to surgeon. However there is general agreement that palatoplasty (soft palate at least) should be performed between 6-12 months of age. Basically there are three groups of palatoplasty techniques. One is for hard palate repair, second for soft palate repair and the third based on the surgical schedule. Hard palate repair techniques are Veau-Wardill-Kilner V-Y, von Langenbeck, two-flap, Aleveolar extension palatoplasty, vomer flap, raw area free palatoplasty etc. The soft palate techniques are intravelar veloplasty, double opposing Z-plasty, radical muscle dissection, primary pharyngeal flap etc. And the protocol based techniques are Schweckendiek's, Malek's, whole in one, modified schedule with palatoplasty before lip repair etc. One should also know the effect of each technique on maxillofacial growth and speech. The ideal technique of palatoplasty is the one which gives perfect speech without affecting the maxillofacial growth and hearing. The techniques are still evolving because we are yet to design an ideal one. It is always good to know all the techniques and variations so that one can choose whichever gives the best result in one's hands. A large number of techniques are available in literature, and also every surgeon incorporates his own modification to make it a variation. However there are some basic techniques, which are described in details which are used in various centres. Some of the important variations are also described.

## INTRODUCTION

Surgical techniques for cleft lip and palate are continuously evolving, more so the techniques of cleft palate repair. The techniques, their variations, the outcome and rehabilitation procedures are very well described in the available literature. This article presents the common surgical techniques presently in vogue and some of their important and common variations. Rehabilitation procedures, management of complications and management of velopharyngeal incompetence (VPI) is out of the scope of this article.

There are a number of preoperative issues to be considered before embarking on palatal surgery irrespective of the technique one chooses. These are as follows:

### Preoperative considerations

#### Schedule of Palatoplasty

The difference of opinion starts right from this stage. The majority accept 6 to 12 months as the optimum age for palatoplasty. There are many centres performing palatoplasty between 12-18 months. There are a few who perform at least a part of the palatoplasty as late as 10-12 years.[1] Ideally, one should consider the development of babbling as an indicator of the time to reconstruct the palate. In developing countries the situation is different. Hence in patients arriving late palatoplasty is performed before lip repair. However, the author advocates palatoplasty before lip repair in all cleft lip and palate children to avoid the dropout and delay in repair of the functionally important palate in these patients.[2]

#### Preoperative Investigations

There is no specific investigation for cleft lip and palate repair. From the point of view of anaesthesia, haemoglobin, body weight, urine albumin, sugar and microscopy are adequate. Some of the centres perform throat swab culture and sensitivity. However, the majority of the cleft surgeons have discontinued throat swab culture as they found it to be a time-consuming investigation which did not change the approach to surgery.

#### Use of Antibiotics

Use of peri-operative antibiotics has not been discussed adequately in the literature. However, it is accepted that the use of peri-operative antibiotics reduces the postoperative complications. Palatoplasty is performed in a clean contaminated surgical field. The child is intubated for a considerable period of time. There is a possibility of postoperative oropharyngeal and respiratory infection. The cleft palate patients may have otitis media secondary to Eustachian tube dysfunction.[3] Considering these facts, it is preferable to use peri-operative antibiotics depending upon the protocol existing in the institute. It is accepted that the use of intraoperative antibiotics is associated with shorter hospitalization and decreased incidence of postoperative fever.[4]

#### Endotracheal Tube

Palate repair being an intraoral surgery, the endotracheal tube (ETT) is an important factor to be considered. The ETT has to be placed orally. The tongue blade of the mouth gag retracts the ETT and is placed against the mandible and the tongue, hence there is a risk of ETT compression between the gag and the mandible. The use of preformed RAE tube (Ring, Adair, Elwin tube) facilitates the placement of the palate mouth gag without causing a kink in the tube. Earlier, the Oxford tube was specifically designed for cleft lip and palate surgery.[5] This has been replaced by RAE tubes. Attempts are being made to use Flexible laryngeal mask (FLMA) in palatoplasty.[6] Use of LMA reduces the possibility of complications during the emergence from general anaesthesia. Many anaesthetists and surgeons are worried about the displacement of LMA during surgery. Kundra *et al.,* mention that there is a short learning curve and Agrawal's modified gag helps while using FLMA during palatoplasty.[6]

#### Mouth gag

Basically, there are two types of palate mouth gags which are commonly in use. Kilner-Dott and Dingman mouth gags. Dingman gag is a little large and relatively heavy but the inbuilt cheek retractors help in intraoral exposure. There are many modifications of the gag presented in the literature, some of them have already been incorporated in the available instruments in the market.[7] The majority of the modifications are aimed at prevention of kinking of the ETT. The author has modified the tongue blade of the gag by incorporating two parallel bars which come in contact with the mandible so that a space is created for housing the ETT without causing compression.[8]

#### Haemostatic Infiltration

Epinephrine (Adrenaline) saline solution in dilution of 1:200,000 is used for infiltration in palate 5-7 min before the surgery. Lignocaine 0.5 mgm/ml added to this solution enhances the vasoconstriction and the haemostasis. Use of a smaller syringe makes the infiltration and hydrodissection easier in the hard palate region.

#### Position of the patient

The patient is placed supine with neck extended either by keeping a pillow or a rolled towel under the shoulder or by inflating a travel pillow already placed under the child.[9] Many surgeons keep the head of the child on their lap and operate. A head ring under the occiput helps in stabilizing the head.

### Objectives of the Cleft Palate Repair

A large number of techniques and their variations are in use in different centres by different surgeons. However, the objectives and principles of every technique remain the same. There are three major objectives of a cleft palate operation.[10] All of these are inter-related:

- To produce anatomical closure of the defect.
- To create an apparatus for development and production of normal speech.
- To minimize the maxillary growth disturbances and dento-alveolar deformities.

#### Principles of Palatoplasty

- Closure of the defect.
- Correction of the abnormal position of the muscles of the soft palate, especially Levator Palati.
- Reconstruction of the muscle sling.
- Retropositioning of the soft palate so much so that during speech the posterior part of the soft palate comes in contact with the posterior pharyngeal wall during speech.
- Minimal or no raw area should be left on the nasal side or the oral surface.
- Tension-free suturing.
- Two-layer closure in the hard palate region and a three-layer closure of the soft palate.

## SURGICAL TECHNIQUES

The surgical techniques of cleft palate repair which are presently practised by different surgeons in various centres are being presented. There are many variations of each of these techniques. However, only a few of them which are most relevant and useful are being presented.

- von Langenbeck's bipedicle flap technique
- Veau-Wardill-Kilner Pushback technique
- Bardach's two-flap technique
- Furlow Double opposing Z-Plasty
- Two-stage palatal repair
- Hole in one repair
- Raw area free palatoplasty
- Alveolar extension palatoplasty (AEP)
- Primary pharyngeal flap
- Intravelar veloplasty
- Vomer flap
- Buccal myomucosal flap

### von Langenbeck technique

In 1861, Bernard von Langenbeck described a method of uranoplasty (palatoplasty) using mucoperiosteal flaps for the repair of the hard palate region. He maintained the anterior attachment of the mucoperiosteal flap to the alveolar margin to make it a bipedicle flap.[11] Originally only the cleft edges were incised, a lateral incision was made, the flap was elevated from the hard palate, the palatine musculature was divided and finally the sutures were applied.

**Figure 1a-c:**
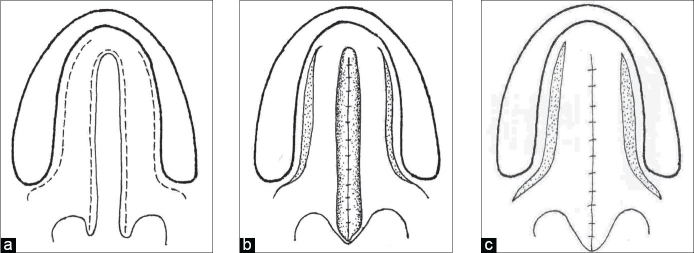
Line diagram of von Langenbeck palatoplasty for an isolated complete cleft palate

This technique is still used in isolated cleft palate repair. The muscle dissection and muscle suturing are done as additional procedures to create a muscle sling[10] [Figure 1].

### Veau-Wardill-Kilner Palatoplasty

Till a few years back this procedure was the commonest technique of palatoplasty. In this technique V-Y procedure is performed so that the whole mucoperiosteal flap and the soft palate are retroposed and the palate is lengthened.[11] However, it leaves an extensive raw area anteriorly and laterally along the alveolar margin with exposed bare membranous bone. The raw area heals with secondary intention. This causes shortening of the palate and results in velopharyngeal incompetence. The raw area adjacent to the alveolar margin also results in alveolar arch deformity and dental malalignment.

To increase the lengthening of the soft palate George Dorrance advocated horizontal back-cut in the nasal lining at the junction of hard and soft palate.[12] This leaves a large raw area on the nasal surface which is left open. This may contract after healing with secondary intention and may undo the palatal lengthening. Since there is single-layer repair in the region of the back-cut, the incidence of palatal fistula is high.

**Figure 2a-d:**
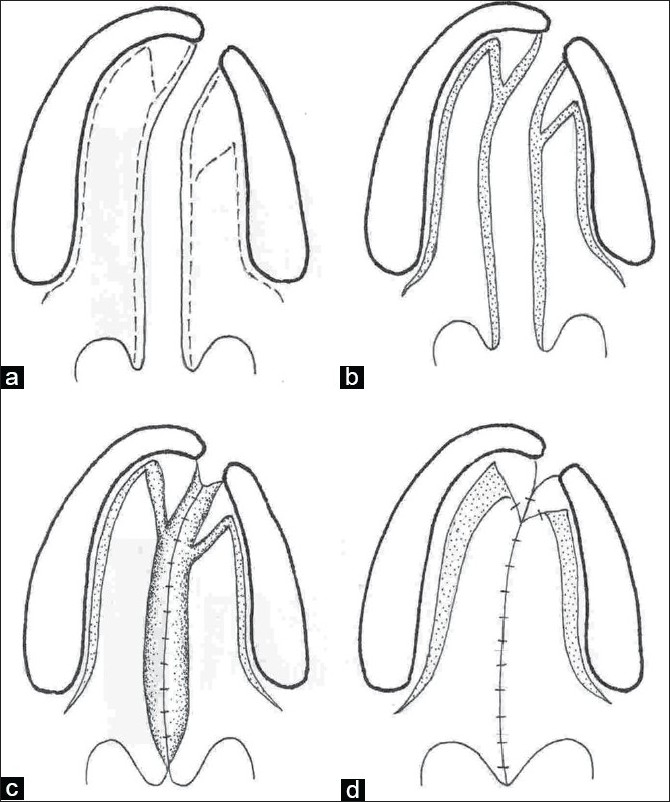
Line diagram showing the Veau-Wardill-Kilner technique of palate repair in a unilateral cleft lip and palate

Because of these drawbacks pushback and V-Y techniques have fallen into disrepute and now less and less centres practise this technique [Figure 2].

### Bardach Two-flap Palatoplasty

**Figure 3a-d:**
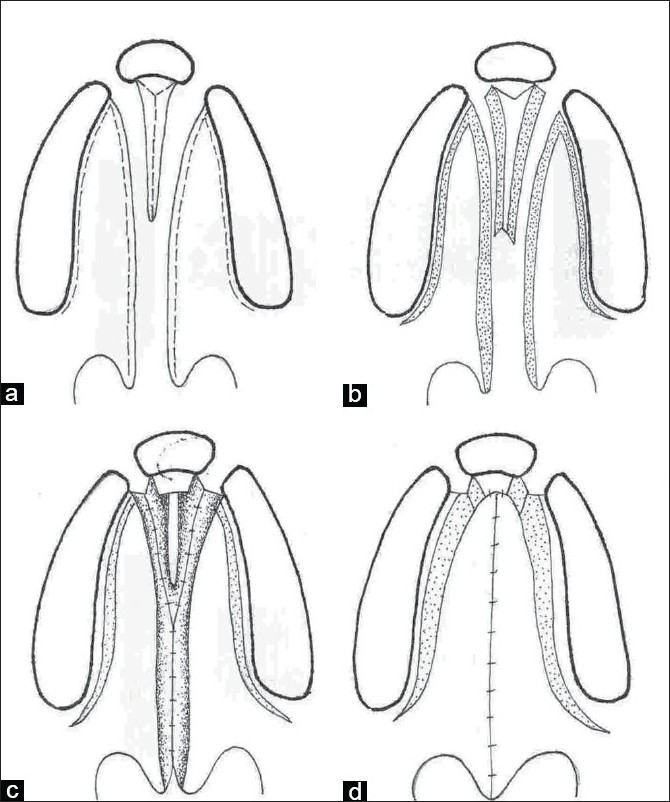
Line diagram showing Bardach two-flap technique of palatoplasty in a bilateral cleft lip and palate

This is a modification of the von Langenbeck technique in which the incision is made along the cleft margin and the alveolar margin. These are joined anteriorly to free the mucoperiosteal flaps.[13,14] These flaps are based on the greater palatine vessels. The soft plate is repaired in a straight line. The levator palati muscle dissection and reconstruction of the muscle sling is performed as in intravelar veloplasty. This is a technique commonly followed presently [Figure 3].

### Furlow Double Opposing Z-Plasty

Furlow adopted a double reverse Z-plasty for the oral and nasal surfaces of the soft palate. The cleft margin forms the central limb. The muscle is incorporated into the posteriorly based triangular flap on the left side for ease of dissection.[15] The hard palate region is closed by making an incision along the cleft margin, elevating the mucoperiosteum from the medial side and taking advantage of the high arch, the cleft is closed in two layers without making a lateral incision. Furlow described the use of the lateral relaxing incision only when necessary.

**Figure 4a-d:**
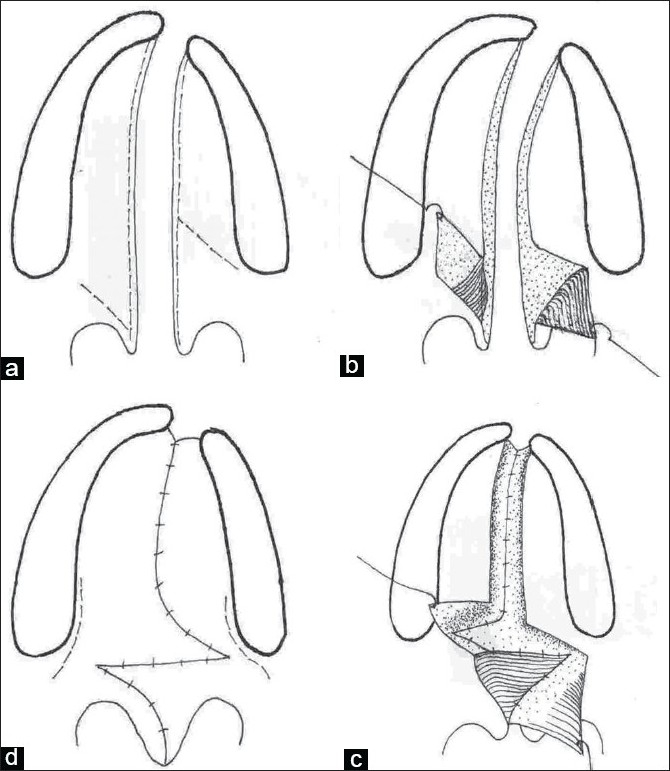
Line diagram showing Furlow Z-plasty technique of palatoplasty in a unilateral cleft lip and palate patient

On transposition of the triangles there is an effective lengthening of the soft palate, the suture line is horizontal and there is good overlap of the levator muscle. Many surgeons claim to have better speech outcome with Furlow repair technique. However, the studies have not proved this objectively. The major objection to the technique is the non-anatomic placement of the muscle [Figure 4].

### Two-stage Palatoplasty

It is a well established fact that unrepaired cleft patients have better maxillary relationship and development. Early palatal surgical intervention causes maxillary hypoplasia. Because of this reason many surgeons used to perform palate repair in two stages. The soft palate was repaired early and later the hard palate was repaired.[16] At the time of introduction of this protocol the soft palate was repaired along with the lip at around four to six months of age and the hard palate was repaired at the age of 10-12 years. This was later reduced to four to five years. This delay significantly reduced the cleft width in the hard palate region and was easy to close without the need for extensive dissection. This reduced the maxillary hypoplasia significantly. However, the speech result was compromised. Hence this technique fell into disrepute. Delaire introduced two-stage functional palatoplasty. A method of cleft palate repair is described, based on a functional repair of the soft palate, followed by closure of the hard palate later taking into account the anatomy and physiology of the palatal mucosa.[17]

### Hole in one repair (One-stage cleft lip and palate repair)

In developing countries repeated hospitalization is a drawback for independent surgery for cleft lip and cleft palate. To avoid this, some of the surgeons popularized a one-stage repair of the full extent of the cleft. This is performed in children above 10 months of age. The surgeons claim extremely good results without any complications.[18,19] This is a good procedure and has gained popularity in our country. This term ‘hole in one’ is borrowed from the Game of Golf and popularised by Prof. K.S. Goleria

### Raw area free palatoplasty

This technique is exactly like the two-flap palatoplasty. Here the palatal lengthening is performed by the nasal mucosa back-cut, however, the raw area is covered with a local flap like the vomer flap or the buccal mucosal flap. On the oral side too an attempt is made to suture all the lateral incisions. This way no raw area is left on either surface.[20] Healing of the palate occurs with primary intention, hence secondary deformities and shortening of the palate is less likely to occur.

### Alveolar Extension Palatoplasty

Michael Carsten recently described alveolar extension palatoplasty (AEP) technique for palatoplasty. In this technique the entire lingual gingivoperiosteal tissue is incorporated into the mucoperiosteal flap. This is expected to lengthen and widen the flap to cover the larger defect. Carsten claims that this procedure is more favourable to angiosomes. This is expected to reduce the maxillary hypoplasia.[21]

### Primary Pharyngeal Flap

To improve the speech in children with cleft palate, primary pharyngeal flap pharyngoplasty is performed in a few centres.[22] Since the majority of these patients will not develop velopharyngeal incompetence after classical palatoplasty, this procedure seems to be an overkill. This creates an abnormal anatomy in all the cleft palate patients, which is not acceptable to most surgeons. This procedure is not popular presently, as it unnecessarily subjects the patients to the disadvantages of pharyngeal flap surgery like sleep apnea, hyponasality etc.

### Intravelar Veloplasty

In 1968 Braithwaite first described the dissection of the Levator Palati from the posterior border of the hard palate, nasal and oral mucosa and posterior repositioning. He described independent suturing of the muscle with that of the opposite side for the reconstruction of the Levator sling.[23] Since then intravelar veloplasty has evolved considerably and many surgeons have modified the surgical details to achieve better anatomical muscle sling reconstruction. Sommerlad advocates radical muscle dissection under a microscope. Sommerlad dissects the levator palate belly separately and sutures independently as the Levator is the dominant muscle for elevation of the soft palate during speech.[24] Court Cutting transects the Tensor Palati and to keep its function intact, the cut end is transfixed with the hook of the hamulus.[25]

During various meetings the discussion on “how much muscle dissection is optimum” remains inconclusive. The majority of the surgeons dissect the muscle but the extent varies. Probably, the end result remains the same.

### Vomer flap

Vomerine mucoperiosteal tissue is very versatile. Most of the surgeons utilize the vomer flap only for repair of the cleft anteriorly in the hard palate region and the alveolar region. The vomer flap in this region is invariably used as a superiorly based turnover flap. This tissue has been revisited and has been extensively used for covering palatal defects. Many varieties of vomer flaps have been described for use in unilateral and bilateral cleft palates for nasal lining and oral mucosa resurfacing.[26]

### Buccal Myomucosal flap

The raw area left over the nasal surface after pushback has always been a matter of concern. Buccal myomucosal flap was used by Mukherjee MM, 1969 to take care of this raw area created after pushback surgery after Veau-Wardill palatoplasty. He had also used bilateral buccal mucosal flaps simultaneously for covering the oral and nasal surfaces.[27] This technique has been recently popularized by Jackson for covering the defect created after back-cut at the junction between hard and soft palate.[28]

### Management of Uvula

Uvula reconstruction is usually not given enough emphasis, however, the parents and patients while assessing cleft palate repair watch the formation of uvula. If uvula is not properly repaired and if it remains bifid the parents are worried and consider it as failure of surgery. Hence one should repair the uvula with great care in two layers. One should use two to three mattress sutures for better approximation of the edges. One should avoid sacrificing the uvula.[29]

### Variations

There are sporadic reports on variations of palatoplasty techniques. To mention a few:

- Bumsted's two-layer closure of palate in very wide cleft palate[30]
- Widmaier-Perko Palatoplasty[31]
- Supraperiosteal dissection of flap in the region of hard palate instead of mucoperiosteal flap[32] This dissection has been advocated so as to minimize the maxillary hypoplasia, however, the surgical dissection in the submucosal plane is bloody, difficult and time-consuming.
- Osada's two-stage palatoplasty[33]
- Frolova primary palatoplasty technique[34]
- Anterior mucoperiosteal hinge for nasal lining in partial cleft palate[35]
- Marginal musculo-mucosal flap[36]

These are some of the variations and procedures which are being performed by a specific surgeon or in a specific centre. The long-term benefits and wider acceptance are awaited.

## Basics of Postoperative Management

### Postoperative feeding

Postoperative oral fluid is given as soon as the child regains full consciousness. Early oral feeding pacifies the child, who then sleeps well.

### Postoperative analgesia

The use of the nonsteroidal anti-inflammatory drug, diclofenac, in the form of rectal suppository provides effective analgesia. In our centre paracetamol suppository and oral suspension are used with satisfactory result. Injectable fentanyl with a basal infusion rate of 0.63 microgram/kg/h is effective in postoperative pain management in children undergoing cleft palate repair. Many centres use parenteral morphine or codeine postoperatively.

### Postoperative arm restraint

Arm restraints are used to avoid self-inflicted trauma with uncontrolled hand movement of the child during postoperative period. But many centres have stopped using arm or hand restraint in these children. These centres report that there is no increase in the complication rate in the absence of these splints.

### Complications

Common complications of any palate surgery are as follows:

### · Immediate complications

Haemorrhage

Respiratory obstruction

Hanging Palate

Dehiscence of the repair

Oronasal fistula formation

### · Late complications

Bifid uvula

Velopharyngeal Incompetence

Abnormal speech

Maxillary hypoplasia

Dental malpositioning and malalignment

Otitis media

### Haemorrhage

Intraoperative haemorrhage is acceptable in the majority of the patients. Use of Epinephrine Lignocaine solution reduces the blood loss. Use of bipolar coagulation proves quite useful in achieving haemostasis. A few patients continue to have bleeding mainly from the bare membranous bony palate and from the edges of the mucoperiosteal flaps. Packing with absorbable compressed gelatin sponge (Gel foam) or oxidized cellulose polymer fibres (Surgicel) are required. The bleeding is more commonly seen in adults and grown-up patients.

In the immediate postoperative period, narrowing of the velopharyneal gap, postsurgical tissue swelling in the soft palate, intrapalatal haematoma, nasal block due to a blood clot and additional bleeding, even if it is minimal, collectively result in respiratory obstruction. If the child is sedated and there is tongue fall, the resulting respiratory problem poses a considerable risk.

### Respiratory obstruction

Postoperative pulse oximetry is mandatory for all palatoplasty patients till the child is fully awake and is able to maintain O_2_ saturation with natural air without any support. In high-risk patients with cleft palate with micrognathia or syndromic cleft, it is safer to apply a tongue suture during the postoperative period for 12-24 hours. Some of the surgeons routinely use tongue suture after palatoplsty in all the patients.

### Hanging Palate

This term has been coined by the author. It is not an uncommon complication after palatoplasty. The anterior wound dehiscence results in the detachment of the mucoperiosteal flap. This complication is very disturbing for the parents as well as the surgeon. An innovative management protocol using a methyl methacrylate obturator fixed to the alveolar arch has been described.[37]

### Oronasal fistula

The oronasal fistula itself is a major subject for discussion. The incidence varies from less than 2% to over 40% in different publications. The common sites are the junction between the hard and the soft palate and the anterior palate region. However, every part of the palatal repair may have breakdown resulting in oronasal fistula. Most of the new variations and additional procedures are aimed at reducing the incidence of fistula formation. The fistula may be significant or insignificant. This depends upon the site and the dimension. Every significant fistula requires repair at an appropriate time. The details are beyond the scope of this article.

## CONCLUSION

Cleft palate surgery has seen major refinements over the past 20-30 years. The attempt to create a normal anatomy has improved the overall outcome of surgery. A team approach has decreased the morbidity and secondary deformities caused by the cleft. The current trend of early palate repair, early assessment with improved instrumentation, early evaluation of velopharyngeal function and the development of procedures for achieving better bony alignment should result in more predictable end results in terms of speech and maxillofacial growth following cleft palate repair.
